# State of knowledge of the relationship between celiac disease and oral pathology: A scoping review

**DOI:** 10.4317/medoral.26950

**Published:** 2025-01-26

**Authors:** Rafael Martín-Masot, Pablo Ramos-García, Encarnación Torcuato-Rubio, María Isabel Pérez-Gaspar, Víctor Manuel Navas-López, Miguel Ángel González-Moles, Teresa Nestares

**Affiliations:** 1 Pediatric Gastroenterology and Nutrition Unit, Hospital Regional Universitario de Málaga, Málaga, Spain; 2 Institute of Nutrition and Food Technology "José Mataix Verdú" (INYTA), Biomedical Research Centre (CIBM), University of Granada, Granada, Spain; 3 Department of Pharmacology and Pediatrics, School of Medicine, University of Málaga, Málaga, Spain; 4 School of Dentistry, University of Granada, Granada, Spain; 5 Biohealth Research Institute, Ibs.Granada, Spain; 6 Dentistry private practice, Malaga, Spain; 7 Department of Physiology, Faculty of Pharmacy, University of Granada, Granada, Spain

## Abstract

**Background:**

Celiac disease (CD) is a systemic disorder characterized by an enteropathy of highly variable clinical expression, in which the relationship with oral pathology has not yet been fully elucidated. We aimed to update the current knowledge on oral manifestations in CD, to identify evidence gaps and to point out future research lines.

**Material and Methods:**

PRISMA-ScR guidelines were followed. MEDLINE/PubMed, Embase, Web of Science and Scopus were searched for primary-level observational studies to analyze the prevalence of oral pathology in CD patients, without language or publication date restrictions.

**Results:**

We included 107 studies, encompassing a total of 26148 celiac patients and 36063 controls. Our results point to several oral pathologies with higher prevalence in CD patients than in healthy controls, most notably recurrent aphthous stomatitis (RAS)(n=69 studies/12606 celiac patients), developmental enamel defects (*n*=61 studies/5037 patients), dental caries (*n*=33 studies/2730 patients), delayed eruption (*n*=12 studies/1062 patients), atrophic glossitis (*n*=10 studies/1062 patients), angular cheilitis (*n*=7 studies/10606 patients), gingivo-periodontal diseases (*n*=7 studies/1122 patients), and Sjögren's syndrome (*n*=5 studies/953 patients).

**Conclusions:**

CD is frequently associated with oral pathologies, including RAS, dental caries, gingivitis, decreased salivary flow, dental enamel defects and some relevant autoimmune processes, such as oral lichen planus and probably Sjögren's syndrome.

** Key words:**Oral pathology, celiac disease, dental caries, recurrent aphthous stomatitis, developmental enamel defects, scoping review.

## Introduction

Celiac disease (CD) is a complex or multifactorial systemic autoimmune pathology, considerably prevalent in the world -1.4% of the general population (1,2) related to gluten consumption in genetically susceptible individuals carrying HLA-DQ2 or HLA-DQ8 haplotypes. CD is characterized by the presence of specific antibodies and enteropathy (3,4); its diverse clinical spectrum ranges from totally asymptomatic cases to more severe cases in which, in addition to the typical intestinal involvement manifestations, there are also symptoms and signs derived from the involvement of other organs and systems (4).

Although the severity and digestive and/or systemic symptoms show great variability between patients (5), the common feature is an exacerbated immune response. The difficult diagnosis of the disease is delayed (6), which could imply that the exacerbated and chronic activation of the immune system remains uncontrolled for longer, leading to a worse prognosis, accentuation of symptoms and, probably, favouring the appearance of other autoimmune diseases, which are frequent in patients with CD (7). The only current treatment for CD is a strict gluten-free diet (GFD) for life.

CD patients present a wide range of diverse pathological processes affecting the oral cavity, whose pathogenic mechanisms are not fully understood (5). Of these, recurrent aphthous stomatitis (RAS) and developmental enamel defects (DED) are the best documented in terms of evidence. Currently, we know that both processes occur at a higher prevalence in CD patients compared to the general population (8,9). However, even though this information is highly relevant, there are knowledge gaps in the management of these disorders. For example, the only meta-analysis that has been published to date (9) regarding RAS focuses on its prevalence in CD and its distribution according to age groups, compared to the general population. However, we know nothing about the pathogenic mechanisms of the disease. RAS is an autoimmune process in the majority of cases (80%) (10) and under this approach, its occurrence in CD could represent a comorbidity of CD with other autoimmune processes, as has been previously reported - autoimmune thyroiditis, Sjögren's syndrome, type I diabetes, among others - (11,12). However, in 20% of cases, RAS is caused by a deficiency of vitamin B12, folic acid or serum iron, and consequently some cases of RAS may be secondary to the essential clinical fact of CD itself, i.e. intestinal malabsorption (10). The question is not trivial since it concerns how to approach the management of these patients, either with replacement therapy or with immunosuppressive treatment of the oral mucosa (13,14). Something similar occurs with DED, for which the two meta-analyses that have been published find a higher prevalence in deciduous teeth and in children, compared to the control groups, although nothing is reported on aetiopathogenic and risk factors (8,9). The scientific literature on oral manifestations in CD contains a large number of primary-level studies regarding several aspects, including dental caries, delayed dental eruption, alterations in salivary flow -essentially hyposalivation-, periodontal disease and Sjögren's syndrome. However, there are no systematic reviews and meta-analyses on these issues, apart from those mentioned for RAS and DED, so the evidence is limited to published case series; although for some of these oral disorders there are a remarkable number of primary-level studies that could allow for research designs that would provide solid evidence. This observation is important because some oral manifestations in the context of CD are very relevant, such as dental caries or periodontal disease, for which we have limited evidence, both in relation to their prevalence, pathogenic mechanisms, risk factors, prevention and treatment.

For these reasons, it was decided to carry out this study, under the design of a scoping review, with the general objective to update the state of knowledge of the oral manifestations that appear in patients with CD - especially with regard to their prevalence, their aetiological and risk factors, as well as their prevention and treatment -, to identify those aspects of the issue that can be meta-analysed, and to point out evidence gaps in the knowledge in order to indicate future lines of research.

## Material and Methods

This scoping review followed the reporting guidelines outlined in the “Preferred Reporting Items for Systematic Reviews and Meta-Analyses extension specifically designed for Scoping Reviews” (PRISMA-ScR) (15).

- Search strategy

MEDLINE/PubMed, Embase, Web of Science and Scopus databases were searched for studies published prior to December-2022. Search strategy was developed by controlling for specific thesaurus terms used by the databases (i.e., MeSH and Emtree) with free terms, designed in order to maximize sensitivity (Supplement 1). Moreover, additional records were explored handsearching the reference lists of the retrieved studies. All references were organized and managed using Mendeley v.1.19.8 (Elsevier, Amsterdam, The Netherlands), and any duplicate references were removed.

- Eligibility criteria

Inclusion criteria: 1) Original primary-level studies, without publication language restriction or date limits; 2) Observational study design; 3) Studies analyzing the prevalence of oral lesions or conditions in patients affected by CD (not being strictly necessary the presence of a control arm), and/or the magnitude of association (with control group, i.e., healthy general population); 4) When data derived from the same sample of patients, it was selected depending on the amount of data provided and year of publication. Name and membership of authors, study’s location, recruitment period and source of patients were scrupulously contrasted to differentiate populations in studies.

Exclusion criteria: 1) Aggregated data for oral and extraoral manifestations; 2) Lack of essential data; 3) No clinico-pathological outcomes; 4) Overlapping populations; 5) Studies performed in animals or *in vitro*; 6) Reviews, meta-analyses, meeting abstracts, editorials, book chapters, letters, case reports, personal comments, erratum or retracted articles.

- Study selection process

Two blinded authors (ETR and RMM) independently applied the eligibility criteria. Discrepancies were then resolved by consensus with a third author (PRG). The records were selected across two subsequent stages. In the stage-I, titles and abstracts were screened looking for potential records meeting inclusion criteria. In the stage-II, the records were read in full text and excluded if eligibility criteria were not met. Initially, the evaluators underwent three rounds of training and calibration, piloting 50 documents in each round, in order to become proficient in the process of identifying and selecting studies. An optimal inter-agreement proportional score (relative frequency of agreement=99.36%) was obtained. The inter-rater reliability was also measured calculating a Cohen’s kappa statistic, obtaining an almost perfect agreement (κ=0.96).

- Data extraction

One author (ETR) systematically extracted data from the included primary-level studies by employing standardized data collection forms within Excel and Word (v.16.66.1, Microsoft. Redmond, WA). Data were gathered on the first author, publication year, study population, sample size, oral diseases, objectives, study design, and key results. These datasets were also subjected to an additional cross-check by two more authors (RMM and PRG), resolving discrepancies by consensus.

- Analysis and evidence synthesis

The present scoping review methodology appears pertinent for the purpose of investigating for evidence-based findings and identifying potential gaps across potential areas where evidence may be lacking. In this sense, the clinical implications of oral diseases in celiac patients, singularly epidemiological data such as prevalence and/or magnitude of association, were investigated across primary-level studies, in order to synthesize current evidence, search for potential evidence gaps, and guide future research. Key results were shown in descriptive Tables, using a systematic methodological approach, and discussed in depth.

## Results

- Results of the Literature Search

The flow diagram (Supplement 2) graphically depicts the search and subsequent process of identification, screening and selection of studies. Overall, 3703 records were retrieved: 1290 from Embase, 1229 from Scopus, 686 from MEDLINE/PubMed, and 498 from Web of Science. After duplicates removal, 1675 records were piloted and screened according to titles and abstracts, leaving a sample of 116 papers for full text evaluation. Finally, 107 studies meeting all eligibility criteria were included were included in the scoping review (all primary-level studies finally included and full-text exceluded -jointly with their corresponding exclusion criteria- are respectively referenced in Supplements 3 and 4). The Table 1 summarizes the characteristics of the 107 selected studies, while Supplement 5 describes in detail the characteristics of our sample, in a study-by-study manner.

- Analysis and evidence synthesis

Oral hard tissues

Dental enamel defects: Most published studies have concluded that in celiac population, DED are more frequent than in healthy population, with prevalence rates varying depending on the study (Supplements 5-8). Notably, DED categorized as systematic of grade I, as per the classification system devised by Aine *et al*., have consistently emerged as the most frequently observed subtype. However, other authors did not find differences between celiac patients and healthy subjects. Furthermore, age-stratified analyses conducted by Aine *et al*. (*p*<0.05) and Cheng *et al*. (*p*<0.001) found higher prevalence of DED in pediatric celiac patients when compared to adults. In addition to age, various other factors have been scrutinized for their potential association with DED in CD, including Anti-transglutaminase antibody levels, HLA genotype, adherence to a GFD, age at initiation of GFD, and histological findings without demonstrating any significant correlations.

Caries: Considerable controversy exists concerning the relationship between dental caries and CD (Supplements 5-8). A substantial portion of studies do not identify differences in prevalence between celiac and healthy subjects, while other groups argue in favor of a higher caries incidence in the healthy population, and some assert a greater prevalence of caries in CD. Housseiny *et al*. did not discern differences in terms of quality of life pertaining to the presence of caries in celiac patients. Regrettably, the presence of caries in the context of celiac disease remains underexplored in relation to variables such as HLA type, adherence to a gluten-free diet, histological characteristics or associated symptoms.

Delayed dental age and delayed eruption: Most of the published studies arrive at the consensus that in CD, dental age is delayed compared to chronological age with an average delay of approximately 15 months, and this delay appears to be more prevalent among individuals with CD than in the general population (Supplements 5-8). Nevertheless, certain authors did not identify significant differences between celiac and healthy subjects in this regard.

Oral soft tissues

Recurrent aphthous stomatitis: The prevalence of recurrent aphthous stomatitis (RAS) appears higher in the celiac population compared to the healthy population (Supplements 5-8), although some studies found no differences. When comparing children vs. adults, Niknam *et al*. and Majsiak *et al*. concurred that RAS is more common in adult celiac patients than in pediatric celiac patients. Cheng *et al*. concluded that RAS is more frequent in patients with DED. Regarding the association between RAS and classical celiac symptoms, Sashi *et al*. supported such a link, while Ertekin *et al*. and Bhattachary found evidence to the contrary. Several studies concluded that RAS episodes improve or disappear after adopting a GFD, except for Zoumpoulakis, and that this improvement is more significant at 12-24 months of GFD compared to the initiation of the diet. Mubarak *et al*. identified a positive association between RAS and elevated anti-transglutaminase antibody levels. Erriu *et al*. initially suggested that having at least one copy of HLA-DQB1*02 was associated with a reduced risk of RAS, but in a subsequent study, they found inconclusive data. Conversely, Majorana *et al*. observed a higher prevalence of the DRw10 and DQw alleles in celiac patients with RAS. Housseiny *et al*. concluded that the quality of life is lower in celiac patients with RAS compared to celiac patients without RAS. Notably, no correlation between RAS and histology has been established.

Xerostomia and salivary flow: All studies examining xerostomia consistently report a higher prevalence in the celiac population compared to the healthy population (Supplements 5-8). Ahmed *et al*. did not find differences in the prevalence of xerostomia between newly diagnosed celiac patients and those with more than one year of GFD adherence. Additionally, Nota *et al*. concluded that celiac patients with hematological or muscular issues are at a higher risk of developing xerostomia. Regarding salivary flow, the literature presents conflicting data. Some authors conclude that individuals with celiac disease have a lower average salivary flow compared to the healthy population, while others assert that it is similar to that of the healthy population. However, Liu *et al*. have found that unstimulated salivary flow is higher in individuals with CD, but stimulated salivary flow was comparable between celiac and healthy individuals.

Angular cheilitis: Two studies support that angular cheilitis is more prevalent in the celiac population than in the healthy population (Supplements 5-8), while two others do not show differences between celiac patients and healthy subjects. Ahmed *et al*. found no differences in the prevalence of angular cheilitis between recently diagnosed celiac patients and those with more than one year of GFD.

Geographic tongue: According to the geographical tongue, Campisi *et al*. found that geographic tongue was more frequent in coeliacs than in the healthy population. (*p*<0.001), but other authors found no differences.

Atrophic glossitis: Campisi *et al*. found that atrophic glossitis was more frequent in coeliacs than in the healthy population (*p*<0.001), but other authors found no differences.

Sjögren syndrome: There are few studies examining the prevalence of Sjögren's syndrome in CD, with conflicting data. Collin *et al*. concluded that SS is more common in celiac patients than in patients with other disorders (*p*=0.01), while Ayar *et al*. found that the prevalence of SS in celiac patients and healthy subjects is similar using the diagnostic criteria of the American College of Rheumatology and the American European Consensus Groups (*p*=0.49).

Periodontitis, gingivitis and other gingival disorders: The literature on these conditions is limited, but most studies agree that there are no differences in periodontal and gingival health between celiac patients and healthy individuals, with the exception of Van Gils *et al*., who concluded that gingival issues were more common in celiac disease than in the healthy population (*p*<0.001).

Other oral manifestations: The relationship between CD and other oral pathologies has been considerably less studied. Regarding dental plaque, only Shteyer *et al*. concluded that the plaque index was higher in newly diagnosed celiac patients compared to healthy individuals and celiac patients on a GFD. Other authors who addressed the topic did not find differences between celiac patients and healthy subjects. Occlusion abnormalities were studied by Marzec-Koronczewska *et al*. and Petrecca *et al*., concluding that 38% and 59%, respectively, of celiac patients required orthodontic treatment. Ertekin *et al*. investigated tooth loss and found no differences between celiac patients and healthy individuals. However, Van Gils *et al*. concluded that tooth loss was more prevalent in celiac patients compared to the healthy population (*p*<0.05). They also identified a higher risk of glossodynia in celiac patients than in healthy individuals (OR = 2.2, 95% CI = 1.2 - 3.9, *p*<0.001), which contrasts with the findings of Shahraki *et al*., who reported no significant differences between celiac and healthy subjects. Liu *et al*. did not find differences in fissured tongue between celiac individuals and healthy subjects. Spijkerman *et al*. observed the presence of oral squamous cell carcinoma in 0.24% of celiac patients, and Tursi *et al*. documented the presence of lupus in 1.3% of celiac patients. On the other hand, Saraceno *et al*. concluded that there were no differences in the prevalence of dental agenesis between celiac patients and healthy individuals.

## Discussion

The selection process of primary-level studies has allowed us to include in this scoping review a total of 107 scientific articles that focus on the presence of oral manifestations in CD. These studies provide information on a total of 26,148 patients with CD. In the following, we describe, in each particular case, the knowledge provided by these studies on the oral disorders presented in CD patients and the knowledge gaps in this regard.

**Figure 1:**
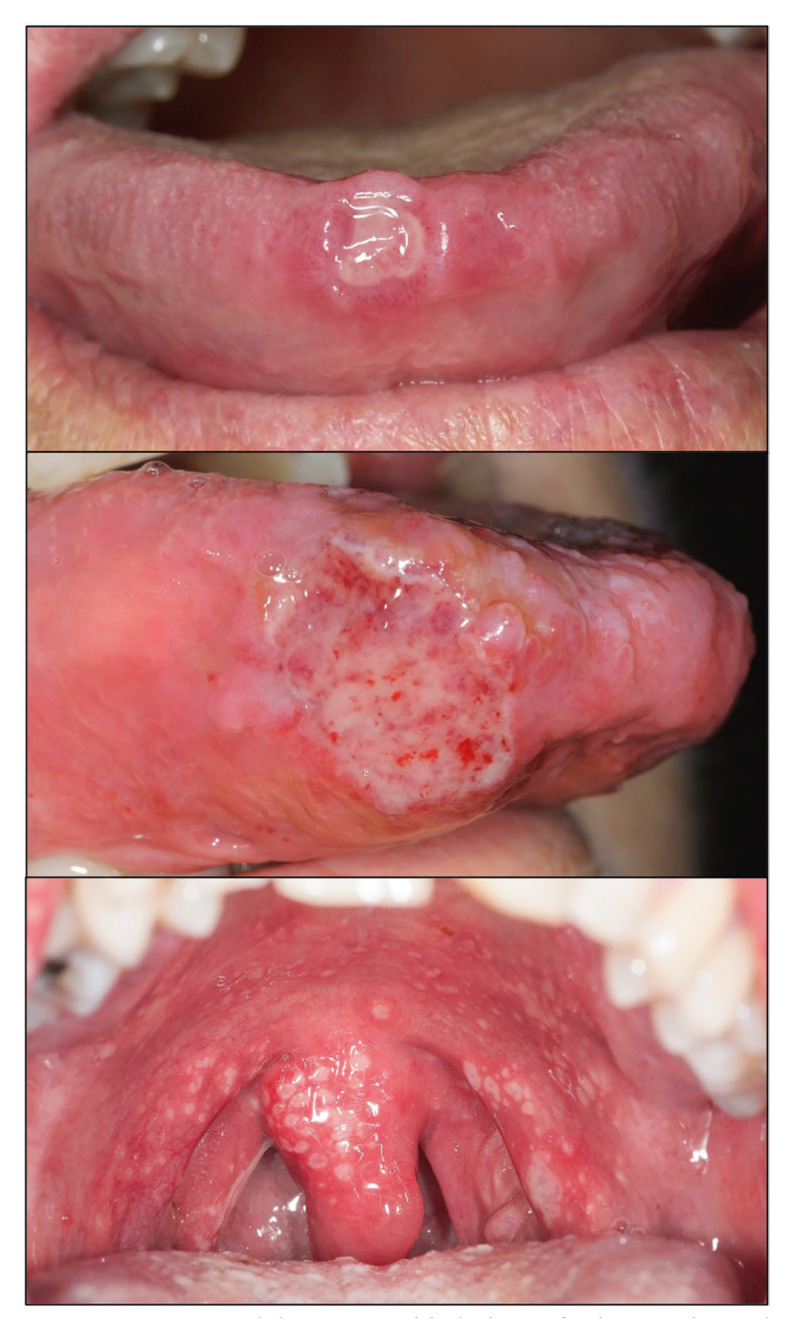
Recurrent aphthous stomatitis lesions of minor, major and herpetiform types.

Recurrent aphthous stomatitis (RAS): RAS is one of the most common oral conditions affecting humans. Review studies estimate the prevalence of RAS to be as high as 36.5% of the population (10,16). RAS usually appears in childhood and adolescence, decreasing in prevalence significantly after the third decade of life (17); it is a disease of unknown aetiology, probably multifactorial, which seems to be due to an essentially autoimmune mechanism; some contributing or triggering factors have been documented such as local trauma, stress and psychological alterations, the menstrual cycle, some nutritional deficiencies or some systemic diseases including chronic inflammatory bowel diseases (ulcerative colitis, Crohn's disease and CD) (18). From a clinical point of view, RAS occurs with the appearance of ulcers on the oral mucosa that present with a relapsing course, in outbreaks that appear with a variable frequency depending on the clinical type. RAS is considered to be one of the most painful conditions affecting the oral cavity, particularly in cases involving the tongue where small ulcerations can cause incapacitating pain. Three clinical types of RAS have been classically described (10), namely: minor forms - two or three oval lesions of no more than one cm in greatest diameter, usually lasting no more than 10 days, affecting essentially the labial mucosa, buccal mucosa, tonsillar pillars or floor of the mouth respecting the attached gingival mucosa and palate; major forms -several deep ulcers, usually larger than one cm in diameter, which cause severe pain, last up to one month, and may cause scarring after healing-, and herpetiform RAS -multiple small, painful ulcerations, resembling herpetic lesions- (Fig. 1). From the point of view of its clinical course, RAS can present in the form of infrequent and spaced out outbreaks, although in other cases the frequency of the outbreaks is such that after the healing of one outbreak, almost immediately or even superimposed, another one begins, with the disease then becoming, in fact, a chronic process that generates enormous physical suffering in the patient (complex or complicated aphthosis)(19).

RAS is one of the most frequent oral manifestations in celiac patients. Our scoping review has selected 69 primary-level studies presenting results regarding RAS in a total of 12,606 CD patients. Based on the descriptive data reported by the studies, 26.4% of CD patients present with RAS, ranging from 0% to 69%, with no substantial differences between the Figures in children (29%; range: 0%-69%) and adults (23%; range: 1.90%-56.3%). Taking into consideration that, in the general population, RAS usually appears in childhood and decreases significantly after the third decade (17), the fact that in CD there are no differences in prevalence between children and adults could indicate that in celiac patients, the pathogenesis of RAS could be linked to the CD itself, in patients in which this process has not been diagnosed, and not to other factors related to RAS that affect the non-celiac population. This idea is also supported by three primary-level studies reporting that a GFD decreases the frequency of RAS. However, an inherent limitation of these studies is that, in addition to their small number, they are observational in nature -two retrospective cohorts and one cross-sectional study- with study designs that provide limited evidence. On the other hand, RAS is one of the few oral conditions that has been studied in coeliac patients based on high-evidence research designs. The only meta-analysis on RAS in CD (9) published in 2017 does not report prevalence data, although it points out that the frequency of developing RAS in celiac patients is 3.79 times higher than in the control population (95% CI = 2.67 - 5.39); this result strongly indicates that, in clinical practice related to CD, attention to the management of RAS should acquire special relevance. However, neither the above-mentioned meta-analysis nor the results of our scoping review provide information about the most relevant aspects of the disease, and the evidence gaps are very relevant. For example, there is no information on whether RAS in celiac patients is a consequence of nutritional deficiencies linked to malabsorption or whether it is due to an autoimmune process concomitant with RAS and, consequently, we do not know which should be the most appropriate treatment (replacement of nutritional deficiencies or topical immunosuppression on the oral mucosa). There is also no information on the clinical presentation of the disease - major, minor, herpetiform forms, number of outbreaks, etc. - and this obviously also limits the treatment plan. Another knowledge gap arises from the lack of information on the potential role and impact that the microbiome could exert on the association between RAS and CD, so future primary-level studies designed for this purpose are required. The meta-analysis published in 2017 (9) was conducted on 20 studies; to date several new primary-level studies have been published on RAS in CD - a total of 69 papers in our scoping review- and although probably not all would be suiTable for meta-analysis, it is very likely that the evidence derived from a new meta-analysis would greatly reinforce the knowledge about this association and its clinical management.

Dental caries: An oral condition that has classically been considered to be particularly prevalent in celiac patients is dental caries, being hypoplastic enamel, changes in salivary composition and low salivary flow factors that could contribute to the development of dental caries in coeliacs (5). This fact has attracted considerable interest due to the negative health consequences of an increased risk of caries associated with CD. The data derived from this scoping review come from 33 primary-level studies addressing this issue, although prevalence data can only be extracted from 6 of them. According to these studies, 49.18% of all celiac patients suffer from dental caries; the reported frequency of dental caries in celiac patients under 18 years of age is 56.33%, while in those over 18 years of age, dental caries has been found in 42.03% of cases. It should be highlighted that there are no systematic reviews and meta-analyses addressing this problem, perhaps because there are not enough primary-level studies on the issue, or because of the heterogeneity with which caries is reported across primary studies (i.e., in terms of incidence, prevalence, DMFT index, DMFS index, etc). However, two meta-analyses published on the prevalence of caries in the general population report Figures of 44% and 48% (20,21) which a priori do not seem to be very different from what has been reported in CD. Therefore, a first approach of interest is to verify whether the prevalence of caries is really higher in celiac patients vs. the population without CD.

While it is recognized that the aetiology of dental caries is multifactorial, celiac patients may suffer from some conditions linked to the disease that could increase the risk of developing dental caries. Firstly, reasons associated with their diet should be considered. Historically it has been assumed that the GFD is nutritionally adequate, but actually the GFD is based on personal choice of foods with the sole condition and restriction of gluten exclusion. Thus, a GFD is characterized by the combination of natural foods that do not contain gluten with industrially produced products that are also gluten-free, but of questionable nutritional quality (22). It is often supplemented with additives, often containing sugar, as flavour enhancers (22). In fact, both in our previous studies (23) as in those of other authors (24), increased consumption of simple sugars in coeliac populations following a GFD has been reported, which contributes to a more cariogenic diet.

**Figure 2:**
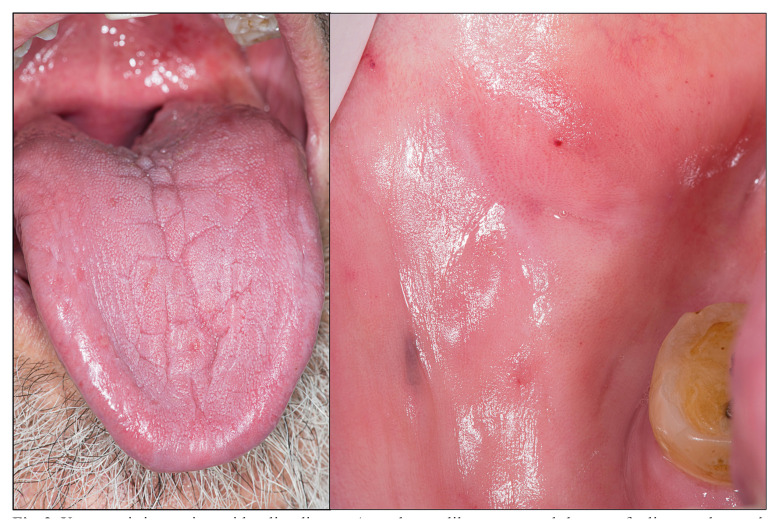
Xerostomia in a patient with celiac disease. A parchment-like mucosa and absence of saliva are observed.

Alterations in salivary flow: A reduced ability to produce saliva has also been frequently reported in celiac patients, with dry mouth (xerostomia) being a major cause of dental caries, probably as a consequence of the acidic environment it generates and the changes towards a more cariogenic oral microbiota linked to a decrease in salivary flow (25). This scoping review has identified 10 primary level-studies focused on the analysis of salivary production capacity in celiac patients, although only 5 of them provide data on frequency. Two primary-level studies analyze saliva production capacity according to the presence of xerostomia, i.e. assessing the appearance of the oral mucosa which, in the absence of saliva, should appear smooth and shiny, parchment-like, probably reddened and, in extreme cases, without saliva on the floor of the mouth (Fig. 2); these papers show xerostomia in 13.95% of cases. Three other primary-level studies address this issue by measuring salivary flow, which is undoubtedly more accurate and reliable. Saliva production capacity is considered to be impaired if the patient produces < 1 ml of saliva per minute, for 15 minutes. Studies analyzing saliva production using this methodology report an average frequency of reduced salivary flow in 39.9% of celiac patients.

Sjögren's syndrome: One possible cause of saliva deficiency in this population is linked to Sjögren's syndrome in CD (26). This syndrome of autoimmune origin presents, among other stigmas, with a progressive autoimmune destruction of the salivary glandular tissue with the consequent xerostomia (26), which causes a high risk of caries in patients, particularly affecting the cervical areas of the teeth (Fig. 2). Consequently, if there really is a higher prevalence of caries in CD and this is due to a lack of saliva, caries should primarily affect the cervical area of the teeth, although there is no information on this in the literature. Our study has identified 5 papers that address the concomitance of Sjögren's syndrome and CD, although only 4 of them give frequency data, which averages 2.63% of cases of Sjögren's syndrome in celiac patients. It is known that the prevalence of the syndrome in the general population ranges between 0.1% and 0.72% (26), from which it is deduced that this syndrome could indeed be more prevalent in CD, presumably due to comorbidity between both pathologies, which would indicate a broader autoimmune status in these patients than that strictly limited to the intestinal mucosa in CD. In any case, more primary-level studies and evidence-based studies in the form of systematic reviews and meta-analyses are needed to confirm these findings.

**Figure 3:**
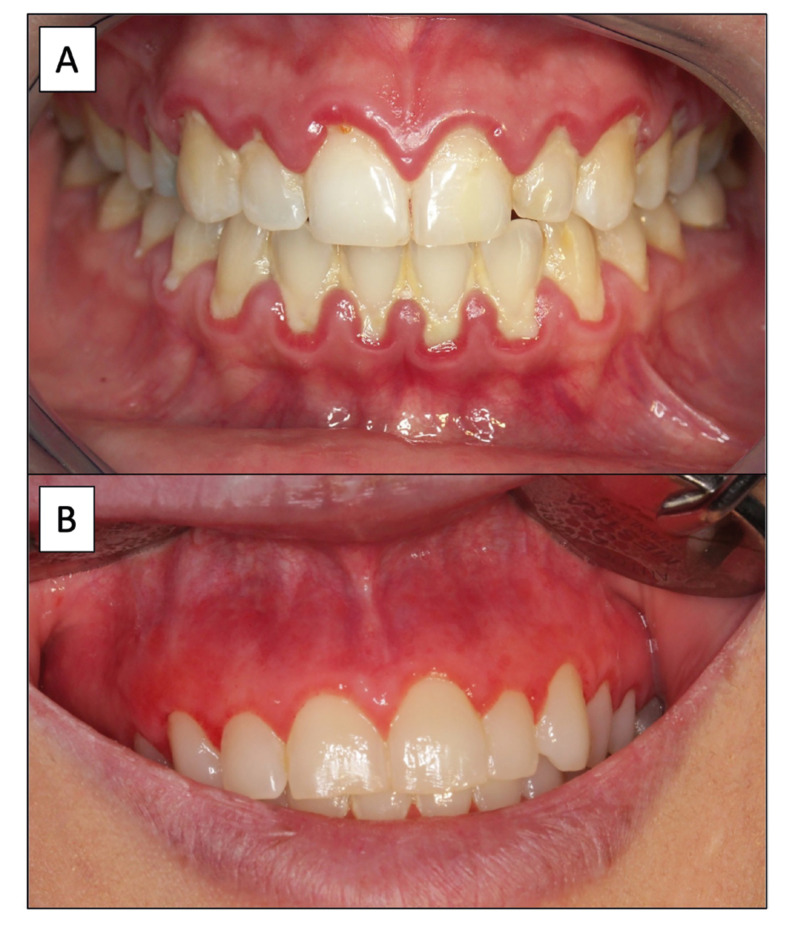
A) Plaque-induced gingivitis in a patient with celiac disease. An inflammatory ridge is observed along the gingival cervical surfaces close to the teeth, and a large amount of bacterial plaque over the teeth; B) Desquamative gingivitis in a patient with celiac disease and oral lichen planus. An intense generalized reddening of the whole gingiva is observed.

Gingival and periodontal diseases:These diseases have also been linked to CD in some case series. Theoretically, some of the alterations of the oral cavity in CD -decreased salivary flow and alterations in the microbiota- could promote the development of gingival and periodontal alterations. However, knowledge of this aspect is drastically limited by the scarce number of primary-level studies on the subject. In relation to periodontitis, only one paper (27) reports that 24% of celiac patients suffer from periodontitis. Bearing in mind that this problem appears in 61.6% of the general population, according to a recent meta-analysis (28), the relationship between periodontitis and CD should be questioned and, in any case, studied in greater depth under homogeneous and consensus-based inclusion criteria in order to allow comparisons. Something similar occurs with gingivitis -an inflammation of the gingiva that initially does not affect the periodontal tissue and which in its most common form is due to the accumulation of bacterial plaque secondary to poor oral hygiene (Fig. 3). Only two papers address the occurrence of gingivitis in CD, with prevalences of 7.59% and 45% respectively. In the absence of meta-analytic studies on the prevalence of gingivitis in the general population, some primary-level studies indicate that the prevalence is very high -over 80% in the North American population (29)-, which in principle seems to question whether gingivitis is a CD-related event; however, this should be a focus of further research. In relation to gingivitis, a special type of gingivitis, called desquamative gingivitis, which reflects the involvement of the gingiva in autoimmune processes, especially oral lichen planus, deserves a special comment (Fig. 3). In this regard, own studies have shown on the basis of evidence that patients with oral lichen planus -a very prevalent oral autoimmune disease (30,31)- suffer from CD in 8.66% of cases (32), with the risk of CD in patients with oral lichen planus being 18.44 times higher than for the general population (*p*=0.005). This indicates that many patients with CD and lichen planus probably develop desquamative gingivitis which, due to its autoimmune nature, deserves special management based on the application of topical immunosuppressive agents. However, it is not known how often oral lichen planus occurs in celiac patients, which is of interest not only because lichen planus deserves specific treatment in these patients -oral topical immunosuppression (33)- but also because currently we know that oral lichen planus carries a significant risk of progression to oral cancer, as also demonstrated by our own studies (34-36).

**Figure 4:**
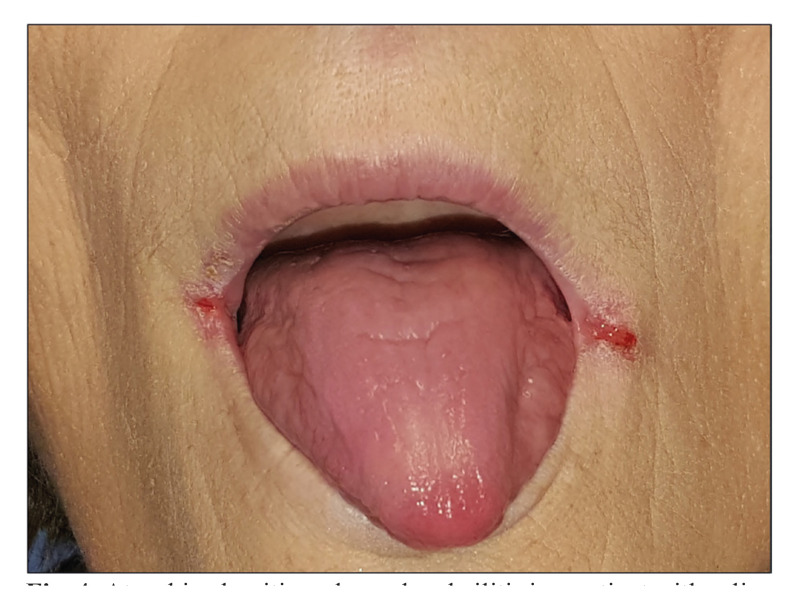
Atrophic glossitis and angular cheilitis in a patient with celiac disease and deficiency of serum iron.

Atrophic glossitis and angular cheilitis: Atrophic glossitis is a lingual condition characterized by the presence of atrophy of the lingual epithelium, especially on the dorsum of the tongue, with disappearance of the filiform and fungiform papillae of the tongue, resulting in discomfort with a shiny, smooth appearance of the tongue (37) (Fig. 4). Ten primary-level studies have focused on atrophic glossitis in CD, although only 8 provide information on its frequency -8.1% of celiac patients-. There is no evidence-based information on the prevalence of atrophic glossitis in the general non-coeliac population, which obviously limits knowledge on the association between both processes which, if significant, would probably be caused by nutritional deficiency, presumably of iron, due to malabsorption. This is not a trivial issue because atrophic glossitis has classically been considered as a lingual precancerous condition (Plummer-Vinson syndrome) (38). A clinical picture of atrophic glossitis could also appear in the context of acute erythematous candidiasis of the oral mucosa (37); taking into account that one of the essential actors in the development of oral candidiasis is xerostomia, we should accept that celiac patients could be significantly predisposed to oral candidiasis, although there is no information on this, and it is certainly a point of interest for future research on CD. Patients with intraoral candidiasis often also develop candidiasis of the labial commissures, which manifests as commissural or angular cheilitis (Fig. 4). Commissural cheilitis in CD has been studied in 7 case series, although only 5 give information on its frequency -6.98% of celiac patients in average develop commissural cheilitis-. It is important to recognize cases of commissural cheilitis caused by candidiasis as there will then necessarily also be intraoral candidiasis that needs to be treated; treatment of angular cheilitis alone with topical antifungals, without treatment of intraoral candidiasis, will inevitably result in recurrence of cheilitis. Angular cheilitis in CD could also be caused by a deficiency linked to malabsorption.

Dental enamel defects: In addition to dental caries, celiac patients may develop dental enamel defects. These defects manifest themselves essentially in the form of changes in enamel translucency, opacities of greater or lesser extent or complete loss of enamel (39). These enamel defects in CD appear to be related to a deficiency in calcium absorption linked to the disease (40). This is known from two evidence based meta-analyses (8,9) which indicate that the prevalence of enamel defects is 50% of celiac patients (95% CI = 44% - 57%), with the risk of developing enamel defects ranging 5.69 times higher in CD vs. non-celiac population. Published meta-analyses on this aspect analyze 18 and 32 primary-level studies respectively (8,9). Our scoping review has identified 61 papers focusing on the study of enamel defects, including 5,037 celiac patients; of these papers, 51 give frequency estimates of enamel defects in 47.42% of CD cases. A new meta-analysis seems pertinent in this respect as there are several new studies that could be included and also the patients in the final sample. This aspect is relevant because, among other reasons, there is a considerable lack of knowledge about how enamel defects can affect other dental pathologies, including the risk of caries, and how their management should be approached.

Delayed dental eruption: CD patients may have delayed eruption of their teeth, which has been linked to growth delay (5). Twelve primary-level studies have been published on this aspect, although only five give Figures on its frequency, which amounts on average to 23.6% of patients (range: 0%-38%); however, there is no solid evidence on the subject.

## Conclusions

Our scoping review shows that CD is frequently associated with certain oral pathologies, including recurrent aphthous stomatitis, dental caries, gingivitis, decreased salivary flow, dental enamel defects, and some relevant autoimmune processes such as oral lichen planus and probably also Sjögren's syndrome, which also behave as potentially malignant oral disorders. Clinicians responsible for the diagnosis and management of patients with CD, as well as patients themselves, should be informed about these associations in order to implement appropriate measures for treatment and follow-up. Furthermore, knowledge should be increased in some aspects of interest, including whether or not GFD can modify the prevalence of occurrence in CD; in this regard, it is important to recognise whether GFD could even increase the prevalence of dental caries in CD as a consequence of the fact that these diets rich in gluten-free products with a high content of simple sugars.

## Figures and Tables

**Table 1:** Summarized study characteristics.

Total sample		107 studies
Year of publication	Range Min (first publication)	1984
	Range Max	2022
Study design	Cohort	70
	Case-control	10
	Cross-sectional	27
Study population (within CD)	Recurrent aphthous stomatitis	69
	Dental enamel defects	61
	Caries	33
	Other oral manifestations (occlusal anomalies, oral squamous cell carcinoma, lupus, loss of teeth, dental plaque, dental agenesis, burning tongue, fissured tongue)	16
	Delayed dental age and delayed eruption	12
	Atrophic glossitis	10
	Salivary flow	10
	Xerostomia	9
	Angular cheilitis	7
	Periodontitis, gingivitis and other gingival disorders	7
	Geographic tongue	6
	Sjögren Syndrome	5

## Data Availability

Data is contained within the article or supplementary material.
